# Prediction of marbofloxacin dosage for the pig pneumonia pathogens *Actinobacillus pleuropneumoniae* and *Pasteurella multocida* by pharmacokinetic/pharmacodynamic modelling

**DOI:** 10.1186/s12917-017-1128-y

**Published:** 2017-07-01

**Authors:** Lucy Dorey, Ludovic Pelligand, Peter Lees

**Affiliations:** Comparative Biological Sciences, Royal Veterinary College, London University, London, UK

**Keywords:** Marbofloxacin, Pharmacokinetic/Pharmacodynamic, *A. Pleuropneumoniae*, *P. Multocida*, Pig, Monte Carlo simulation, Time-kill curves

## Abstract

**Background:**

Bacterial pneumonia in pigs occurs widely and requires antimicrobial therapy. It is commonly caused by the pathogens *Actinobacillus pleuropneumoniae* and *Pasteurella multocida*. Marbofloxacin is an antimicrobial drug of the fluoroquinolone class, licensed for use against these organisms in the pig. In recent years there have been major developments in dosage schedule design, based on integration and modelling of pharmacokinetic (PK) and pharmacodynamic (PD) data, with the objective of optimising efficacy and minimising the emergence of resistance. From in vitro time-kill curves in pig serum, PK/PD breakpoint Area under the curve (AUC) _24h_ /minimum inhibitory concentration (MIC) values were determined and used in conjunction with published PK, serum protein binding data and MIC distributions to predict dosages based on Monte Carlo simulation (MCS).

**Results:**

For three levels of inhibition of growth, bacteriostasis and 3 and 4log_10_ reductions in bacterial count, mean AUC_24h_/MIC values were 20.9, 45.2 and 71.7 h, respectively, for *P. multocida* and 32.4, 48.7 and 55.5 h for *A. pleuropneumoniae.* Based on these breakpoint values, doses for each pathogen were predicted for several clinical scenarios: (1) bacteriostatic and bactericidal levels of kill; (2) 50 and 90% target attainment rates (TAR); and (3) single dosing and daily dosing at steady state. MCS for 90% TAR predicted single doses to achieve bacteriostatic and bactericidal actions over 48 h of 0.44 and 0.95 mg/kg (*P. multocida*) and 0.28 and 0.66 mg/kg (*A. pleuropneumoniae*). For daily doses at steady state, and 90% TAR bacteriostatic and bactericidal actions, dosages of 0.28 and 0.59 mg/kg (*P. multocida*) and 0.22 and 0.39 mg/kg (*A. pleuropneumoniae*) were required for pigs aged 12 weeks. Doses were also predicted for pigs aged 16 and 27 weeks.

**Conclusions:**

PK/PD modelling with MCS approaches to dose determination demonstrates the possibility of tailoring clinical dose rates to a range of bacterial kill end-points.

**Electronic supplementary material:**

The online version of this article (doi:10.1186/s12917-017-1128-y) contains supplementary material, which is available to authorized users.

## Background

Marbofloxacin is a synthetic third-generation fluoroquinolone, developed for sole veterinary use. It has high bioavailability when administered to pigs by intramuscular injection [1]. It accumulates in the cytosol of macrophages, leucocytes, neutrophils, epithelial lining fluid and plasma [2, 3]. Marbofloxacin, as a lipid-soluble organic acid and low to moderate plasma protein binding, achieves good tissue penetration and a high volume of distribution [4]. Concentrations in the lung, liver and kidney exceed those in plasma. However, concentrations in the biophase, the pulmonary epithelial lining fluid in pigs with pneumonia, at steady state will be determined by free drug concentration in plasma. This has been shown to be 50.6% of total concentration in pigs [4].

Lees and Aliabadi [4] reported that marbofloxacin exerts a prolonged post-antibiotic (PAE) and sub-minimum inhibitory concentration (MIC) PAE effects. It has a broad spectrum of antibacterial activity, is bactericidal and exerts a concentration-dependent killing action [5]. The antimicrobial spectrum includes Brucella spp. and Mycoplasma spp. Marbofloxacin is licensed for treatment of pneumonia caused by the pig pneumonia pathogens, *Actinobacillus pleuropneumoniae*, *Pasteurella multocida* and *Streptococcus suis* [6]. Bacterial pneumonia in pigs is also caused by other organisms, including *Bordetella bronchiseptica* and *Mycoplasma hyopneumoniae*.

Over the last two decades, there have been major advances in designing dosage schedules of antimicrobial drugs, based on integration and modelling of pharmacodynamic (PD) and pharmacokinetic (PK) data. These approaches have provided novel strategies for predicting drug dosages that optimise efficacy and minimise opportunities for the emergence and subsequent spread of resistance [6–15]. Optimising dosage may involve reducing doses which may be too high as well as increasing doses when they are too low.

Many authors have proposed that, for fluoroquinolones as a group, the integrated PK/PD indices correlating with successful therapeutic outcome are maximum plasma concentration (C_max_)/MIC and area under plasma concentration-time curve (AUC_24h_)/MIC ratios [5, 7, 8, 11, 16, 17].

The PD parameter most commonly used in establishing the potency of antimicrobial drugs is MIC, the lowest concentration based on two-fold dilutions, inhibiting visible bacterial growth after 24 h incubation under standard conditions (Clinical and Laboratory Standards Institute (CLSI) M31-A3) [18]. Most laboratories use the internationally recommended CLSI guidelines to ensure standardisation [13]. However, these guidelines have limitations regarding accuracy, because they involve the use of doubling dilutions, giving potential for up to 100% error on single isolate estimates. For many purposes, this is acceptable, and indeed is necessary, when large numbers of isolates are to be examined. However, it may not always be appropriate when MIC is correlated with PK data as a basis for PK/PD breakpoint determination. Therefore, based on methods previously described [19–21] five overlapping sets of two-fold dilutions were used in this study to reduce inaccuracy from up to 100% to no more than 20%. The mutant prevention concentration (MPC) was used as an additional indicator of potency to MIC; it is defined as the lowest drug concentration required for preventing the growth of the least susceptible cells present in high density bacterial populations.

A second consideration is that CLSI and European Union Committee on Antimicrobial Testing (EUCAST) methods of MIC determination require the use of broths formulated to optimize the growth of each species of bacteria. Therefore, there is almost universal use of internationally recognised guidelines, methods and standards for MIC determination. However, when the objective of potency determination is prediction of dosage for clinical efficacy, based on PK/PD breakpoints, conditions should be representative of in vivo pathological circumstances. Zeitlinger et al. [22] commented that “bacteria with appropriate and well-defined growth in the selected medium should be employed” and “in order to be able to extrapolate data from various models to in vivo situations, models should always attempt to mimic physiological conditions as closely as possible”.

Whilst serum is not identical to extravascular infection site fluids, it is likely to be closer in chemical composition to the biophase than broths, and indeed in immunological constituents also [22, 23]. A comparison of broth MICs with potency determined in biological fluids is therefore relevant to PK/PD breakpoint estimation. For some drugs and pathogens, calculation of a scaling factor to bridge between broth and serum MICs may be warranted [7, 15, 23, 24]. In this investigation MICs and time-kill data were generated for marbofloxacin in pig serum. They were thus determined with reasonable accuracy and in a biological matrix.

**Fig. 1:**
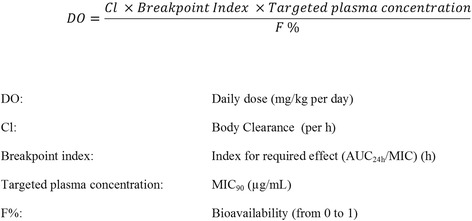
Formula for calculation of the daily antimicrobial drug dose based on PK and PD variables

With the objectives of: extending the therapeutic life of older antimicrobial drugs; ensuring their prudent use and; minimising the emergence of resistance, there have been many proposals to re-evaluate dose schedules that were set, in many instances, prior to the application of PK/PD breakpoints [8–11, 13–15, 19]. For example, Mouton et al. [9] have described the EUCAST approach to dosage re-evaluation. In summary, these authors have proposed that a sound approach to setting dose schedules is to link PK parameters and variables with appropriate indices of potency, applying the general equation for systemically acting drugs (Fig. 1) [19, 25, 26].

The aims of this study were: (1) to integrate published PK variables for the pig with MIC data for pig pathogens obtained in our laboratory in order to generate values of the three PK/PD parameters, C_max_/MIC, C_av_/MIC and T > MIC, for *A. pleuropneumoniae* and *P. multocida*; (2) to model data from time-kill studies of *A. pleuropneumoniae* and *P. multocida*, using multiples of MIC over the range 0.25–8.0 MIC, in order to generate PK/PD breakpoint values of AUC_24h_/MIC for three levels of bacterial kill, bacteriostasis, bactericidal and 4log_10_ reduction in inoculum count; (3) to use published PK data and PK/PD breakpoints, with serum protein binding data and literature MIC distributions, in Monte Carlo simulations (MCS) to predict dose schedules required for: (a) bacteriostatic and bactericidal levels of kill; (b) for 50 and 90% Target Attainment rate (TAR); and (c) for single dosing and daily dosing at steady state.

## Methods

### Minimum inhibitory concentration

Twenty isolates each of *A. pleuropneumoniae* and *P. multocida* were obtained from EU cases of pig pneumonia. These were screened for ability to grow logarithmically in broths and pig serum. Of those exhibiting logarithmic growth in both matrices, MICs in broth were determined by microdilution using two-fold dilutions. From the sensitive isolates, six of each species were selected and MICs re-determined, using artificial broths (Cation Adjusted Mueller Hinton broth for *P. multocida* and Columbia broth for *A. pleuropneumoniae*) in accordance with CLSI guidelines, except that five sets of overlapping 2-fold serial dilutions of marbofloxacin were prepared, as described by Dorey et al. [27]. In addition, the guidelines were adapted to additionally use pig serum in place of broth to enable comparison of the two matrices.

### PK/PD breakpoint determination

**Fig. 2:**
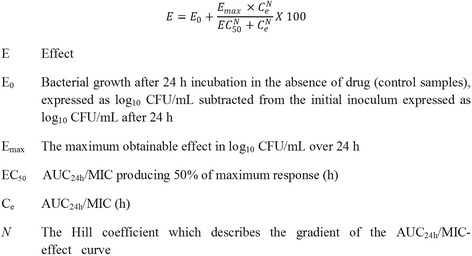
The sigmoidal Emax equation used to model time-kill data by non-linear regression [27]

For the six isolates each of *A. pleuropneumoniae* and *P. multocida*, eight marbofloxacin concentrations, as multiples of MIC, ranging from 0.25 to 8xMIC, were used in time-kill studies over 24 h incubation periods. Determinations were made separately in serum and broth (CB for *A. pleuropneumoniae* and CAMHB for *P. multocida*), as previously described [27, 28]. Each test was repeated in triplicate for the six isolates of each species in both growth matrices. The time-kill curves established bacteriostatic, bactericidal and 4log_10_ reductions in count at 24 h; based on the sigmoidal E_max_ equation (Fig. 2) the data were modelled to provide AUC_24h_/MIC breakpoint values for these three levels of growth inhibition.: E = 0, bacteriostatic, that is 0log_10_ reduction in CFU/mL after 24 h incubation; E = −3, bactericidal, 3log_10_ reduction in CFU/mL; and E = −4, 4log_10_ reduction in bacterial count.

### Dose prediction

#### Deterministic approach

**Fig. 3:**
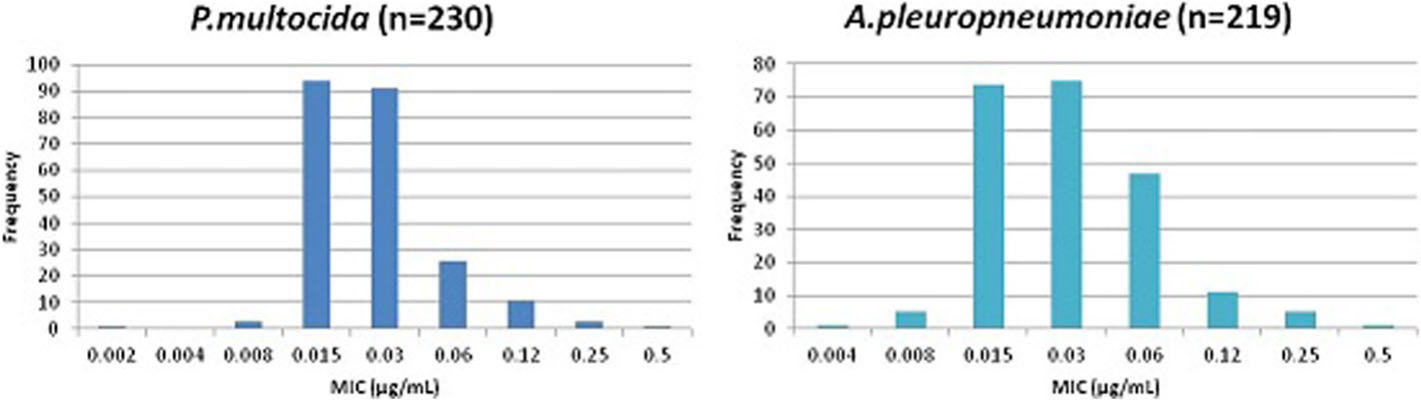
Marbofloxacin MIC distributions of *P. multocida* (*n* = 230) and *A. pleuropneumoniae* (*n* = 219) indicating frequency of MICs. MIC data were generated using CLSI guidelines by de Jong et al. [29]). All isolates were from European countries

For dose prediction using the deterministic approach, mean PK values were obtained from Schneider et al. [1] and PK/PD breakpoint values were used from the present study, together with MIC_90_ values for *P. multocida* and *A. pleuropneumoniae* for marbofloxacin obtained from de Jong et al. [29] (Fig. 3).

#### Monte Carlo simulation

As the PK/PD index that best predicts efficacy for marbofloxacin is the ratio AUC_24h_/MIC, the equation used to calculate dose was:

$$ {\  Dose}_{\left( per\  day\right)}=\frac{Cl\kern0.5em \times \kern0.75em \frac{AUC_{\left(24 h\right)}}{MIC_e}\kern0.75em \times \kern0.5em {MIC}_{distribution}}{f_u\kern0.75em \times \kern0.75em  F} $$

where Cl = body clearance per h, AUC_(24h)_/MIC_e_ (in h) = ratio of experimentally determined area under the serum concentration-time curve over 24 h to the MIC (MIC_e_) of the experimental isolates – six per species - (i.e. the PK/PD breakpoint), MIC_distribution_ = distribution of MICs from epidemiological literature [29], f_u_ (from 0 to 1) = fraction of drug not bound to serum protein and F = bioavailability (from 0 to 1). Thus, calculation of dose depends on: (1) assessment of both PK (Cl, F, f_u,_) and PD (MIC) properties; and (2) determination of an appropriate breakpoint value of the AUC_(24h)_/MIC ratio. This Equation is appropriate for determining daily dosage once steady state has been reached. However, the calculated dose when the initial drug concentration in serum is zero, is very likely to be higher than the dose determined for maintaining the steady state concentration.

**Fig. 4:**
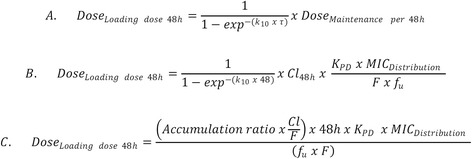
Formulae for calculation of the loading dose for 48 h duration of action, where eq. **a** can be expressed as eq. **b** and simplified as eq. **c**. K_10_ = elimination rate constant; τ = dosing interval in h; Cl_48_ = body clearance over 48 h; K_PD_ breakpoint = AUC divided by 24; MIC_Distribution_ = MICs determined from epidemiological surveys; F = bioavailability (from 0 to 1); f_u_ = fraction of drug not bound to protein binding

Loading doses were calculated for three time periods, 0–24, 0–48 and 0–72 h [7]; the formulae for calculation of dosage for a 48 h period are presented in Fig. 4.

All dosages were computed using Monte Carlo simulation in Oracle Crystal Ball (Oracle Corporation, Redwood Shores, CA, USA), for TAR of 50 and 90%. The probabilities of distribution for the dosage estimation were run for 50,000 simulated trials. Data input comprised: 1) marbofloxacin whole body clearance scaled by bioavailability; clearance data were obtained from the equation Clearance = Dose/AUC, for pigs of three ages [27, 16 and 2 weeks]; 2) drug binding to serum protein [27]; 3) AUC_24h_/MIC breakpoints derived from time-kill curves; and 4) MIC field distribution data [1] (Fig.3). For the MIC field distributions, values were corrected using the serum:broth MIC ratio, as the reported MIC literature values were determined in broth.

## Results

### Minimum inhibitory concentrations

Geometric mean MIC serum:broth ratios for *P. multocida* and *A. pleuropneumoniae* (six isolates of each species) were 1.12:1 and 0.79:1, respectively. For MPC, the ratios were 1.32:1 and 0.99:1, respectively. These did not differ significantly from unity. However, as free drug concentration in pig serum was previously shown to be 50.6% (Dorey et al. 2016b) [28] and as protein bound drug is microbiologically inactive, the corrected ratios, fraction unbound (fu) serum:broth MIC were 0.50:1 and 0.40:1, and 0.67:1 and 0.50:1 for MPC, indicating small (in microbiological terms) but significantly greater potency of marbofloxacin in serum than in broth.

### PK/PD breakpoint values

**Fig. 5:**
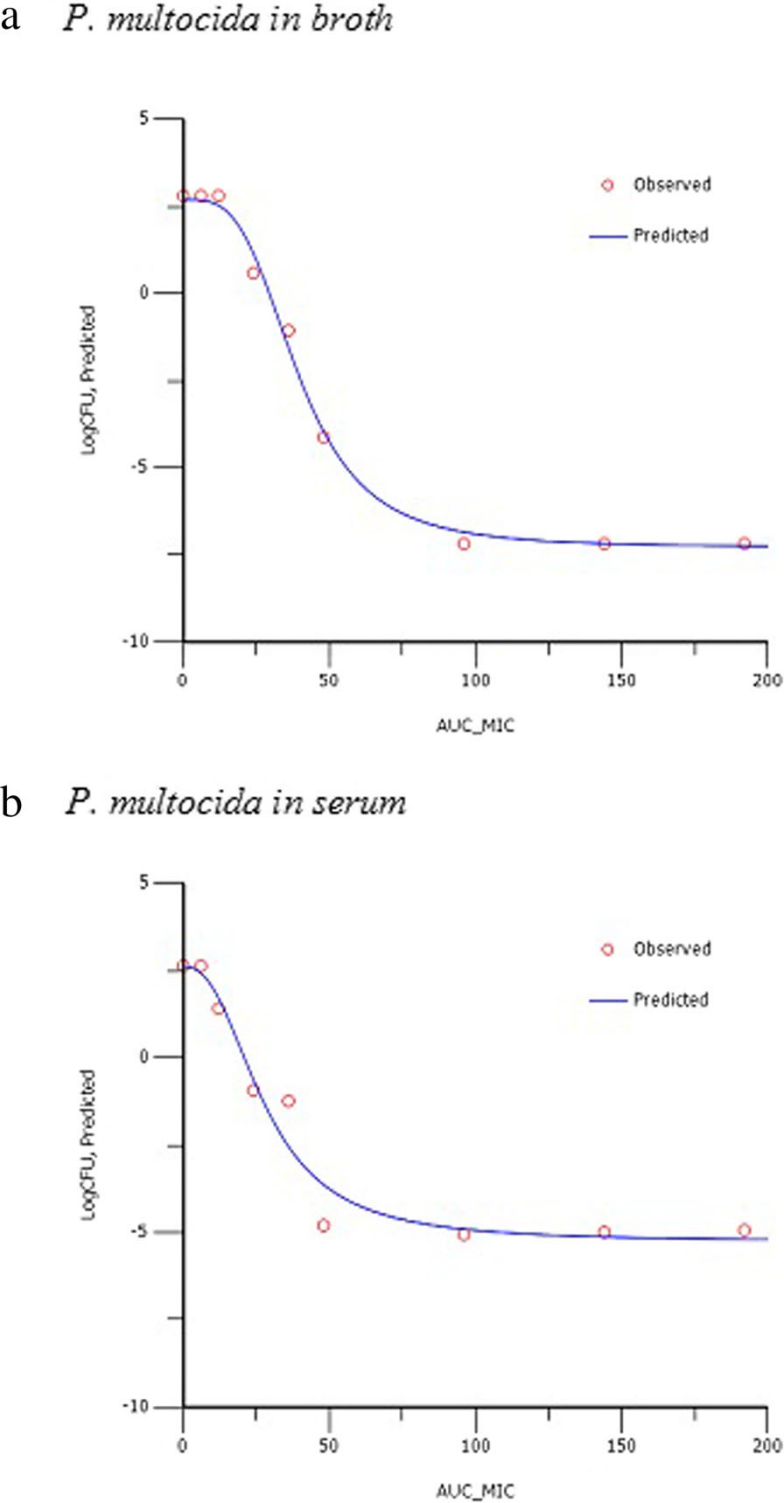
Example plots of AUC_24h_/MIC (h) versus change in bacterial count (log_10_ CFU/mL) for *P. multocida* in (**a**) broth and (**b**) serum. The points represent observed values and the curves are lines of best fit

For each isolate of each organism, growth inhibition curves were generated in the time-kill studies in broth and pig serum. Examples are presented in Fig. 5.

From the time-kill curves, PK/PD breakpoints were derived and are presented in Tables 1 (*P. multocida*) and 2 (*A. pleuropneumoniae*). For three levels of inhibition of growth, bacteriostasis and 3 and 4log_10_ reductions in bacterial count, mean AUC_24h_/MIC values were 20.9, 45.2 and 71.7 h, respectively, for *P. multocida* for serum. Corresponding broth values were 26.5, 41.8 and 48.9 h. For *A. pleuropneumoniae* in serum, breakpoints were 32.4, 48.7 and 55.5 h. and for broth values were 24.8, 42.0 and 54.0 h. Differences between broth and serum, at each level of kill, were relatively small, despite differences between broth MICs and serum MICs corrected for drug binding to serum protein. This is not unexpected, as each inhibition curve was derived using MIC multiples for each fluid.

**Table 1:** *P. multocida* PK/PD modelling of *in vitro* marbofloxacin time-kill curves (mean, standard deviation, *n* = 6)

Parameter (units)	Broth		Serum	
	Mean	SD (CV%)	Mean	SD (CV%)
Log E_0_ (CFU/mL)	2.66	0.26	2.70	0.32
Log E_max_ (CFU/mL)	−7.28	0.37	−5.65	1.43
Log E_max_ - Log E_0_ (CFU/mL)	−9.95	0.11	−8.35	1.11
Gamma	3.26	1.48	1.95	0.23
AUC_24h_/MIC for bacteriostatic action (h)	26.5	4.65 (17.5)	20.9	2.31(11.1)
AUC_24h_/MIC_50_(h)	38.0	8.05 (21.2)	30.5	5.39 (17.7)
AUC_24h_/MIC for bactericidal action (h)	41.8	7.79 (18.7)	45.2	5.58 (12.3)
AUC_24h_/MIC for 4 log10 reduction (h)	48.9	10.8 (22.0)	71.7	34.0 (47.4)

E0 = difference in number of organisms (CFU/mL) in control sample in absence of drug between time 0 and 24 h; E_max_ = difference in number of organisms (CFU/mL) in presence of marbofloxacin between time 0 and 24 h; AUC_24_/MIC_50_ = concentration producing reduction in count to 50% of the E_max_; Gamma = slope of the curve; detection limit = 33 CFU/mL

As a measure of inter-isolate variability, coefficients of variation were determined. These were small to moderate in magnitude (Tables 1 and 2).

**Table 2:** *A. pleuropneumoniae* PK/PD modelling of in vitro marbofloxacin time-kill curves (mean, standard deviation, *n* = 6)

Parameter (units)	Broth		Serum	
	Mean	SD (CV%)	Mean	SD (CV%)
Log E_0_ (CFU/mL)	2.65	0.42	1.79	0.64
Log E_max_ (CFU/mL)	−5.57	0.80	−6.71	0.65
Log E_max_ - Log E_0_ (CFU/mL)	−8.23	0.38	−8.50	0.01
Gamma	3.18	1.20	4.07	0.77
AUC_24h_/MIC for bacteriostatic action (h)	24.8	1.79 (7.2)	32.4	5.30 (16.4)
AUC_24h_/MIC_50_(h)	31.8	4.09 (12.9)	45.6	9.45 (20.7)
AUC_24h_/MIC for bactericidal action (h)	42.0	4.34 (10.3)	48.7	6.58 (13.5)
AUC_24h_/MIC for 4 log10 reduction (h)	54.0	7.04 (13.0)	55.5	6.43 (11.6)

E0 = difference in number of organisms (CFU/mL) in control sample in absence of drug between time 0 and 24 h; E_max_ = difference in number of organisms (CFU/mL) in presence of marbofloxacin between time 0 and 24 h; AUC_24_/MIC_50_ = concentration producing reduction in count to 50% of the E_max_; Gamma = slope of the curve; detection limit = 33 CFU/mL

Dividing the AUC_24h_/MIC ratios by 24 yields concentrations, as MIC multiples, producing bacteriostatic, bactericidal and 4log_10_ reductions in count; these are the K_PD_ values. Numerical values were, respectively, 1.10, 1.74 and 2.04 for *P. multocida* in broth and 0.87, 1.88 and 2.99 for this organism in serum. Corresponding values for *A. pleuropneumoniae* were 1.03, 1.75 and 2.25 (broth) and 1.35, 2.03 and 2.31 (serum).

### Dose determination at steady state (deterministic approach)

Clearance and bioavailability data for healthy pigs were obtained from the literature [1]. Table 3 indicates predicted doses required, at steady state, to achieve three levels of kill. Bactericidal levels of kill were obtained with doses 0.41 mg/kg/day for *P. multocida* and 0.26 mg/kg/day for *A. pleuropneumoniae*.

**Table 3:** Predicted once daily doses calculated by deterministic approach

Level of kill	Predicted daily doses (mg/kg)	
	*P. multocida*	*A. pleuropneumoniae*
Bacteriostatic	0.19	0.15
Bactericidal	0.41	0.26
4log_10_ count reduction	0.65	0.32

### Dose determination by Monte Carlo simulation

Monte Carlo simulations were conducted using: (1) the distribution of clearance around the standard deviation, assuming normal distribution (Schneider et al. 2014) [1]; (2) the distribution of MICs for wild type organisms [29]; (3) free drug fraction in serum; (4) breakpoint values of AUC_24h_/MIC from time-kill studies.

Predicted doses at steady state for 50% and 90% TAR and three levels of kill are presented in Table 4. Doses for bactericidal action for *P. multocida* were 0.43, 0.45 and 0.59 mg/kg, respectively, for pigs aged 27, 16 and 2 weeks. Corresponding values for *A. pleuropneumoniae* were somewhat lower, 0.29, 0.30 and 0.39 mg/kg. The small numerical differences with age of pigs reflect small differences in their PK profiles. Differences in predicted dose for the two bacterial species reflect the differing distributions of MIC of wild type organisms.

**Table 4:** Predicted once daily doses of marbofloxacin at steady state in pigs of three ages (weeks): 27 (A), 16 (B) and 12 (C)^a^

	Predicted daily doses (mg/kg)	Target Attainment Rate	
		50%	90%
A.			
*P. multocida*	Bacteriostatic	0.08	0.19
	Bactericidal	0.18	0.43
	Virtual eradication	0.28	0.67
*A. Pleuropneumoniae*	Bacteriostatic	0.07	0.17
	Bactericidal	0.12	0.29
	4log_10_ reduction	0.15	0.36
B.			
*P. multocida*	Bacteriostatic	0.09	0.21
	Bactericidal	0.20	0.45
	Virtual eradication	0.32	0.72
*A. pleuropneumoniae*	Bacteriostatic	0.08	0.17
	Bactericidal	0.13	0.30
	4log_10_ reduction	0.17	0.37
C.			
*P. multocida*	Bacteriostatic	0.13	0.28
	Bactericidal	0.28	0.59
	Virtual eradication	0.45	0.94
*A. pleuropneumoniae*	Bacteriostatic	0.10	0.22
	Bactericidal	0.18	0.39
	4log_10_ reduction	0.22	0.48

^a^Monte Carlo simulation to achieve 50 and 90% target attainment rates at steady state for three levels of bacterial kill

Table 5 indicates calculated doses for single doses of marbofloxacin administered intramuscularly with three durations of effect, three levels of kill and TARs of 50 and 90%. For a bacteriostatic action of 24 h duration and 50% TAR, predicted doses were 0.12 and 0.03 mg/kg for *P. multocida* and *A. pleuropneumoniae,* respectively. For a TAR of 90%, a bactericidal level of kill and an action over 72 h, the predicted doses were 1.31 and 0.92 mg/kg, respectively, for *P. multocida* and *A. pleuropneumoniae.* Even for a 4log_10_ reduction in count and 90% TAR, predicted doses were relatively low, 2.08 mg/kg (*P. multocida)* and 1.14 mg/kg (*A. pleuropneumoniae*).

**Table 5:** Predicted doses of marbofloxacin for three durations of activity (24, 48 and 72 h)^a^

		Target Attainment Rate			
		*P.multocida*		*A.pleuropneumoniae*	
Dose duration	Level of bacterial kill	50%	90%	50%	90%
0-24 h	Bacteriostatic	0.12	0.29	0.03	0.07
	Bactericidal	0.27	0.64	0.05	0.12
	4 log10 reduction	0.43	1.01	0.06	0.14
0-48 h	Bacteriostatic	0.19	0.44	0.16	0.39
	Bactericidal	0.40	0.95	0.28	0.66
	4 log10 reduction	0.64	1.51	0.34	0.82
0-72 h	Bacteriostatic	0.26	0.60	0.22	0.53
	Bactericidal	0.56	1.31	0.38	0.92
	4 log10 reduction	0.88	2.08	0.47	1.14

^a^Monte Carlo Simulation to achieve 50 and 90% target attainment rates for two levels of bacterial kill and three action durations

## Discussion

### Pharmacokinetics, pharmacodynamics and PK/PD integration

Integration of in vitro generated potency estimates with in vivo PK data has been used extensively to generate three indices to predict clinical outcome, namely the ratios, C_max_/MIC and AUC_24h_/MIC and T > MIC, the time for which concentration exceeds MIC. Integration of pharmacokinetic and pharmacodynamic data for MPC are presented in the Additional file 1. All MPC ratios were much lower than the MIC ratios. From previous marbofloxacin studies, C_max_/MIC and AUC_24h_/MIC ratios provided good correlation with bacteriological cure in human patients [30, 31]. For fluoroquinolones used in veterinary medicine, a C_max_/MIC of 8–10 and an AUC_24h_/MIC greater than 100–125 h have been proposed [13]. However, other studies have suggested that a ratio of AUC_24h_/MIC of 35–50 is effective for Gram-positive bacteria [32]. Many authors have proposed achieving numerical values of AUC_24h_/MIC of 125:1 or 250:1 for gram negative organisms, corresponding to average concentrations over the dosing interval of 5.2 to 10.4, respectively, as a multiple of MIC.

For marbofloxacin, de Jong et al. [29] reported identical MIC_50_ values for both *A. pleuropneumoniae* and *P. multocida* of European pig origin of 0.03 μg/mL and identical MIC_90_ values of 0.06 μg/mL. Schneider et al. [1] reported on marbofloxacin PK in healthy pigs, aged 12, 16 and 27 weeks, administered intramuscularly at three dose rates of 4, 8 and 16 mg/kg. PK/PD integration of data from these studies is presented in a Additional file 1 to this paper. Briefly, for both bacterial species and pigs aged 27 weeks, C_max_/MIC_90_ ratios were 56, 105 and 258, respectively, for marbofloxacin doses of 4, 8 and 16 mg/kg. Even average concentrations over the 92 h period after dosing provided C_average_/MIC_90_ ratios of 9.6, 19.7 and 39.1 for these dose rates. Therefore, a preliminary prediction of likely successful clinical outcome for doses of marbofloxacin in the range 4–16 mg/kg can be made for pig pneumonia caused by the pathogens, *A. pleuropneumoniae* and *P. multocida.*

### PK/PD modelling and breakpoint determination

PK/PD integration is not a precise tool; it should be regarded as a first initial step in predicting efficacy in clinical use. It is especially useful when correlated with outcome in clinical trials. However, the next essential step is to define PK/PD breakpoints for each antimicrobial drug acting against representative isolates of each pathogenic species. PK/PD modelling describes the whole sweep of the concentration-effect relationship. Therefore, any pre-determined level of activity, ranging from bacteriostasis to virtual eradication, indicated by the breakpoint AUC_24h_/MIC index, can be determined. Applying PK/PD breakpoints for indices such as AUC_24h_/MIC, derived from PK/PD modelling, with MCS provides an approach to dose prediction, which takes account of animal species based PK, wild-type MIC distributions, protein binding and breakpoints for specific bacterial species. From such time-kill studies, numerical values of PK/PD breakpoints have been determined by PK/PD modelling by previous workers [7–10, 12, 13, 21, 25, 33].

In this study, breakpoint values for each level of growth inhibition, 0log_10_, 3log_10_ and 4log_10_ reductions in count, were broadly similar for the two growth matrices. This is not unexpected because, although MICs in broth and serum differed, the breakpoint values are based on MIC multiples. AUC_24h_/MIC ratios were similar for broth and serum for each level of kill, being based on MIC values separately for each matrix. Moreover, inter-isolate variability in PK/PD breakpoint values was small to moderate.

### Dosage prediction

PK/PD breakpoints were used with wild type MIC distributions of susceptible pathogens and literature PK data, to conduct MCS to predict doses providing a range of pre-determined levels of kill. The deterministic approach provided an estimate of once daily doses at steady state. It is based on MIC_90_ for each pathogen and average values for other variables. It provides an initial indication of likely effective dosage, but does not take account either of variability or incidence of each input variable and in this study. Predicted daily doses were less than 0.5 mg/kg for a bactericidal kill against both pathogens. Nevertheless, the deterministic approach comprises an initial indication, prior to estimation of population doses for each selected TAR. The latter is a dose encompassing a given percentile of the target population, for example, 50 or 90% and for three pre-determined levels of bacterial kill and for both a single dose and a daily dose at steady state. Monte Carlo simulations predict doses which allow for incidence within MIC distributions and encompass best, worst and all intermediate values for distributions of Cl/F and breakpoint AUC_24h_/MIC ratios. Furthermore, basing potency estimates on serum as a growth matrix, as in this study, has greater relevance to disease conditions than MICs determined in broths. Nevertheless, it is recognised that serum, although preferred to broth for MIC determination, is similar but not identical in composition to the biophase at infection sites, for example pulmonary epithelial lining fluid.

As discussed by Martinez et al. [8] it is the exposure achieved after the first dose, which is most relevant in determining therapeutic outcome. In this study, low marbofloxacin doses were predicted for a greater than bactericidal action, with 90% TAR in both species; for 72 h and a 4log_10_ reduction in count, the predicted doses were 2.08 and 1.14 mg/kg, respectively, for *P. multocida* and *A. pleuropneumoniae*. Both dosages are less than the dosages of 4, 8 and 16 mg/kg studied by Schneider et al. [1]. To achieve a bactericidal action (3log_10_ reduction in count) for *P. multocida* for 90% TAR, once daily doses at steady state were even lower, 0.43 mg/kg for *P. multocida* and for *A. pleuropneumoniae* 0.29 mg/kg for pigs aged 27 weeks.

These predicted doses are lower than those of 2.5 mg/kg and 8 mg/kg investigated by Ding et al. [3] and Ferran et al. [34] as well as the 2 mg/kg recommended dose for several licensed marbofloxacin products. Ferren et al. [34] suggested that even lower doses of marbofloxacin could potentially eradicate low counts (10^5^ CFU/mL) in the lung, while having a minimal impact on the microbiota of the large intestine. On the other hand, Vallé et al. [35] validated for the bovine pneumonia pathogens, *P. multocida* and *Mannheimia haemolytica*, the concept of a single high dose of marbofloxacin compared to a daily dose of 2 mg/kg for 3–5 days. A bactericidal effect against bovine *P. multocida* was achieved within one hour, when marbofloxacin was administered at five times the recommended daily dose (10 mg/kg).

The present study illustrates the principles of using MCS to predict dosages of marbofloxacin for the treatment of pneumonia in the young pig. The proposed dosage regimen is for *A. pleuropneumoniae* and *P.multocida* induced pneumonias only. For other organisms, independent PK/PD studies will be required. However, in future studies it will be important to extend the present findings for *A. pleuropneumoniae* and *P.multocida* also. Whilst the inter-isolate variability in PK/PD breakpoint values for bacteriostatic and bactericidal levels of kill was small in the present study, estimates were based on only six isolates for each species. Moreover, the time-kill studies generating the PK/PD breakpoints used fixed drug concentrations (eight multiples of MIC) for a fixed time period. In clinical use, on the other hand, plasma drug concentrations first increase and then decrease after intramuscular dosing, exposing organisms to a continuously variable concentration. A third consideration is the relatively small number of isolates in the report of de Jong et al. [29].

In future studies, these concerns could be addressed by increasing numbers of isolates in field distribution studies and in PK/PD breakpoint estimation studies. Moreover, exposure of organisms to varying drug concentrations could be addressed by use for example of hollow fibre methods to simulate in vivo patterns of change in concentration with time. Furthermore, marbofloxacin PK data were used as mean and standard deviation values from the literature. In future studies, it will be useful to incorporate individual animal PK data in the MCSs, and, in particular it will be helpful to use population PK data obtained in clinically ill pigs. Finally, the methodology in this study did not consider the contribution to pathogen elimination by the body’s natural defence mechanisms, which are known to be important in immune competent clinical subjects. In addition, potentially beneficial properties of antimicrobial drugs, such as immunomodulatory and anti-inflammatory actions are of importance for some drug classes. Finally, dose prediction studies, as reported in this manuscript, should always be correlated with clinical and bacteriological outcomes in animal disease models and clinical trials [7, 11–13].

## Conclusions

Predicted doses for marbofloxacin for treatment of respiratory tract infections in the pig, caused by *P. multocida* or *A. pleuropneumoniae*, were determined by generating PK/PD breakpoints for several levels of kill, based on modelling PK and PD data. These breakpoint values were used with published MIC distribution data [29] and PK data [1] to determine dosages for three levels of kill, and for once daily doses at steady state and for single doses with both 50% and 90% target Attainment Rates. The findings illustrate the value and principle of using Monte Carlo simulation for determination of optimal doses for a range of outcomes.

## Additional file

Additional file 1: PK/PD integration data. (DOCX 7 kb)

## Abbreviations

CAMHB: Cation-adjusted Mueller Hinton Broth
CLSI: Clinical and Laboratory Standards Institute
EUCAST: Committee on Antimicrobial Susceptibility Testing
MCS: Monte Carlo Simulation
MHB: Mueller Hinton Broth
MIC: Minimum inhibitory concentration
PD: Pharmacodynamics
PK: Pharmacokinetics
TAR: Target Attainment Rate
