# Supplementary material for: Prediction of marbofloxacin dosage for the pig pneumonia pathogens Actinobacillus pleuropneumoniae and Pasteurella multocida by pharmacokinetic/pharmacodynamic modelling
Source: BMC Vet Res. 2017 Jul 1;13:209. doi: 10.1186/s12917-017-1128-y (PMC5493866; doi:10.1186/s12917-017-1128-y)
Supplement: Additional file 1: — PK/PD integration data. (DOCX 7 kb) [file 12917_2017_1128_MOESM1_ESM.docx]

**Supplementary file**

PK/PD integration data ^A, B.^

| A. |  |  |  |  |  |  |
| --- | --- | --- | --- | --- | --- | --- |
|  | C_max_/MIC | | AUC/MIC | | C_av92_/MIC | |
|  | MIC_50_ | MIC_90_ | MIC_50_ | MIC_90_ | MIC_50_ | MIC_90_ |
| 4 mg/kg | 112.7 | 56.3 | 1886.7 | 943.3 | 19.98 | 9.99 |
| 8 mg/kg | 210.0 | 105.0 | 3833.3 | 1916.7 | 41.09 | 20.54 |
| 16 mg/kg | 516.7 | 258.3 | 7600.0 | 3800.0 | 81.57 | 40.78 |
|  |  |  |  |  |  |  |
| B. |  |  |  |  |  |  |
|  | C_max_/MIC | | AUC/MIC | | Cav_92_/MIC | |
|  | MIC_50_ | MIC_90_ | MIC_50_ | MIC_90_ | MIC_50_ | MIC_90_ |
| 4 mg/kg | 185.0 | 92.5 | 2663.3 | 1331.7 | 36.8 | 18.4 |
| 8 mg/kg | 195.3 | 97.7 | 3533.3 | 1766.7 | 48.4 | 24.2 |

^A^ Integration of MIC_50_ and MIC_90_ values, 0.03 and 0.06 µg/mL, respectively, for both *A. pleuropneumoniae* and *P. multocida* [31] and PK data from healthy pigs [1] aged 27 weeks at three dose rates of 4, 8 and 16 mg/kg.

^B^ Integration of MIC_50_ and MIC_90_ values, 0.03 and 0.06 µg/mL, respectively, for both *A. pleuropneumoniae* and *P. multocida* [31] and PK data from healthy pigs [1] aged 12 and 16 weeks at dose rates of 4 and 8 mg/kg.

*P. multocida* PK/PD integration data ^A, B.^

| A. |  |  |  |  |  |  |
| --- | --- | --- | --- | --- | --- | --- |
|  | C_max_/MPC | | AUC/MPC | | C_av92_/MPC | |
|  | Broth | Serum | Broth | Serum | Broth | Serum |
| 4 mg/kg | 5.93 | 4.51 | 99.30 | 75.47 | 1.05 | 0.80 |
| 8 mg/kg | 11.05 | 8.40 | 201.75 | 153.33 | 2.16 | 1.64 |
| 16 mg/kg | 27.19 | 20.67 | 400.00 | 304.00 | 4.29 | 3.26 |
|  |  |  |  |  |  |  |
| B. |  |  |  |  |  |  |
|  | C_max_/MIC | | AUC/MPC | | Cav_92_/MPC | |
|  | Broth | Serum | Broth | Serum | Broth | Serum |
| 4 mg/kg | 9.74 | 7.40 | 140.18 | 106.53 | 1.93 | 1.47 |
| 8 mg/kg | 10.28 | 7.81 | 185.96 | 141.33 | 2.55 | 1.94 |

^A^ Integration of MIC_50_ and MIC_90_ values, 0.03 and 0.06 µg/mL, respectively, for both *A. pleuropneumoniae* and *P. multocida* [31] and PK data from healthy pigs [1] aged 27 weeks at three dose rates of 4, 8 and 16 mg/kg.

^B^ Integration of MIC_50_ and MIC_90_ values, 0.03 and 0.06 µg/mL, respectively, for both *A. pleuropneumoniae* and *P. multocida* [31] and PK data from healthy pigs [1] aged 12 and 16 weeks at dose rates of 4 and 8 mg/kg.

*A. pleuropneumoniae* PK/PD integration data ^A, B.^

| A. |  |  |  |  |  |  |
| --- | --- | --- | --- | --- | --- | --- |
|  | C_max_/MPC | | AUC/MPC | | C_av92_/MPC | |
|  | APP | PM | APP | PM | APP | PM |
| 4 mg/kg | 0.27 | 0.27 | 4.46 | 4.49 | 0.05 | 0.05 |
| 8 mg/kg | 0.50 | 0.50 | 9.07 | 9.13 | 0.10 | 0.10 |
| 16 mg/kg | 1.22 | 1.23 | 17.98 | 18.10 | 0.19 | 0.19 |
|  |  |  |  |  |  |  |
| B. |  |  |  |  |  |  |
|  | C_max_/MIC | | AUC/MPC | | Cav_92_/MPC | |
|  | APP | PM | APP | PM | APP | PM |
| 4 mg/kg | 0.44 | 0.44 | 6.30 | 6.34 | 0.09 | 0.09 |
| 8 mg/kg | 0.46 | 0.47 | 8.36 | 8.41 | 0.11 | 0.12 |

^A^ Integration of MIC_50_ and MIC_90_ values, 0.03 and 0.06 µg/mL, respectively, for both *A. pleuropneumoniae* and *P. multocida* [31] and PK data from healthy pigs [1] aged 27 weeks at three dose rates of 4, 8 and 16 mg/kg.

^B^ Integration of MIC_50_ and MIC_90_ values, 0.03 and 0.06 µg/mL, respectively, for both *A. pleuropneumoniae* and *P. multocida* [31] and PK data from healthy pigs [1] aged 12 and 16 weeks at dose rates of 4 and 8 mg/kg.
